# Rare Hematological Disease of Paroxysmal Nocturnal Hemoglobinuria With Profound Implications for a Gastroenterologist: A Case Report and Literature Review

**DOI:** 10.7759/cureus.8941

**Published:** 2020-07-01

**Authors:** Swetha Parvataneni, Tagore Sunkara, Vinaya Gaduputi

**Affiliations:** 1 Internal Medicine, Geisinger Health System, Lewistown, USA; 2 Internal Medicine, Mercy Medical Center, Des Moines, USA; 3 Internal Medicine, St. Barnabas Hospital - SBH Health System, Bronx, USA

**Keywords:** portal vein thrombosis, eculizumab, paroxysmal nocturnal hemoglobinuria (pnh)

## Abstract

Paroxysmal nocturnal hemoglobinuria (PNH) is a rare type of thrombophilia and hematopoietic stem cell disorder characterized by mutation of the X-linked PIG-A gene. Patients with PNH present either with clinical features of intravascular hemolysis or thrombosis. Visceral vein thrombosis is associated with increased mortality risk. Here, we present an extremely rare case of a young man presenting with extensive thrombosis of multiple visceral veins from PNH.

## Introduction

Paroxysmal nocturnal hemoglobinuria (PNH) is a rare hematologic disorder with 0.5 - 1.5 million cases worldwide [1]. It is an acquired form of hemolytic anemia caused by a somatic mutation in the X-linked PIG-A gene. PIG-A is responsible for synthesizing the glycosylphosphatidylinositol (GPI) that adheres numerous proteins to plasma cell membranes. Mutations in PIG-A result in the deficiency of complement inhibitory proteins CD55 and CD59. In the alternative pathway of complement activation, complement C3 spontaneously hydrolyzes and forms C3 convertase leading to activation of C3 and C5, which results in the formation of a membrane-associated complex, a key step in the terminal pathway. Normally, CD55 controls the level of C3 by degrading C3 convertase, and CD59 inhibits membrane attack complex (MAC) formations. Deficiency of these complement components in PNH results in two major clinical features: intravascular hemolysis and thrombosis [2]. Thrombosis is associated with increased mortality in PNH patients [3]. Hepatic and cerebral veins are the most common sites involved in PNH [4]. Other veins such as the portal vein and visceral veins have also been reported. The involvement of the portal vein and visceral veins without the involvement of hepatic veins is very rare. We present the case of a young male who presented with an extensive portal and visceral vein (superior mesenteric and bilateral renal vein) thrombosis.

## Case presentation

**Figure 1:**
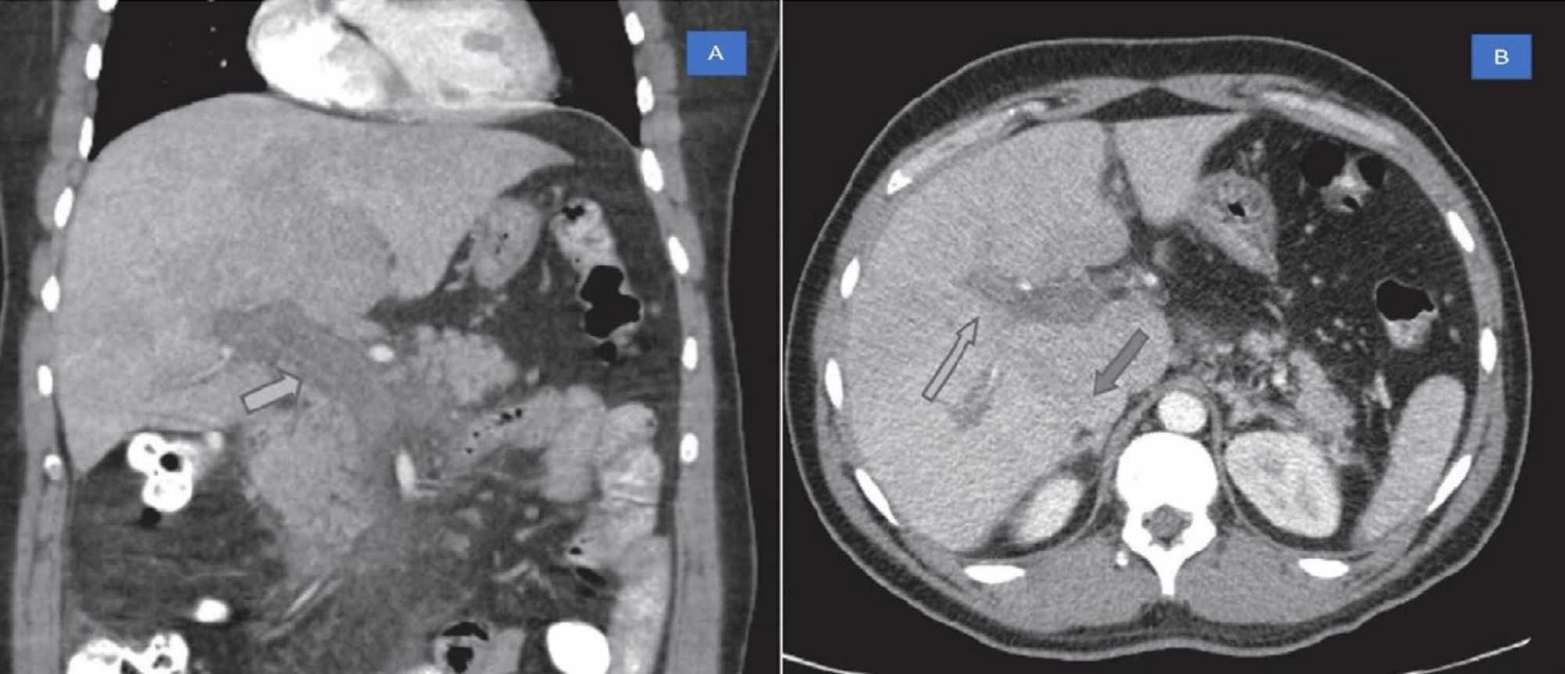
CT scan A: CT demonstrating filling defect within the portal vein representing portal vein thrombosis (PVT) (shown by arrow). B: PVT in the main portal vein and left portal vein (shown by arrows).

A 36-year-old Hispanic male with no significant past medical history presented to the emergency department with four days of diffuse abdominal pain. The patient also reported weight loss of 5 lbs. over the preceding month with generalized fatigue and weakness. Initial lab studies showed normocytic normochromic anemia (hemoglobin [Hb] 8.8 g/dL, mean corpuscular volume [MCV] 88 fL), thrombocytopenia, leukopenia, and mild transaminitis. Anemia work up showed vitamin B12 level of 100pg/ml (190 -950 pg/ml), iron 56 mcg/dl, total iron-binding capacity (TIBC) 291mcg/dl, ferritin 272 ng/ml, increased serum lactate dehydrogenase (LDH) and decreased serum haptoglobin. The patient was found to have elevated urobilinogen levels in urine, suggesting intravascular hemolysis. A computerized CT scan showed extensive portal vein thrombosis, superior mesenteric vein thrombosis, and bilateral renal vein thrombosis (Figure 1).

**Figure 2:**
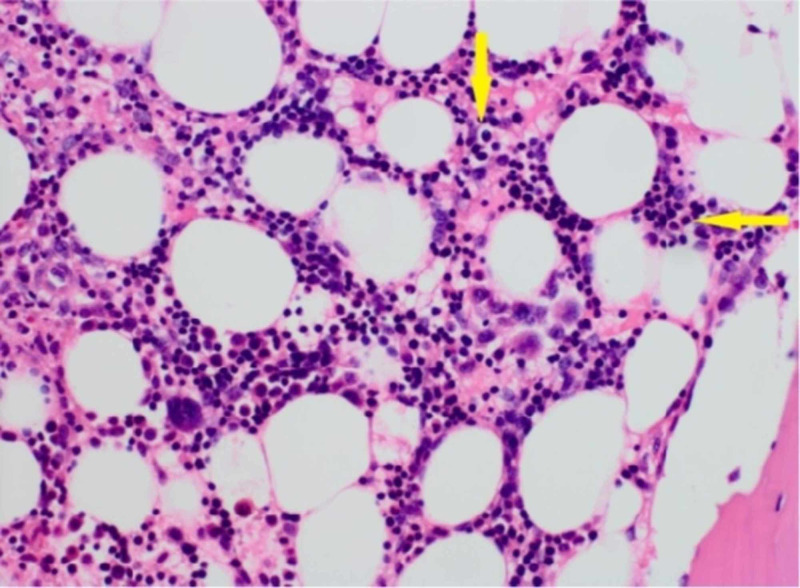
Bone marrow biopsy Bone marrow demonstrating hypercellular marrow with trilineage hematopoiesis with erythroid hyperplasia.

Flow cytometry and bone marrow biopsy confirmed the diagnosis of PNH (Figure 2).

The patient underwent esophagogastroduodenoscopy to screen for esophageal varices. No esophageal varices were found. The patient was started on anticoagulation and eculizumab. 

## Discussion

Abdominal vein thrombosis is a rare, life-threatening disorder that commonly involves the hepatic veins, portal veins, and mesenteric veins. Hepatic vein and portal vein thrombosis are most frequently involved sites in superficial vein thrombosis, commonly seen in the patients with cirrhosis or liver malignancy and uncommon causes include myeloproliferative disorders, PNH, factor V Leiden deficiency, protein C, protein S, anti-thrombin III deficiency, and anti-phospholipid [5]. Approximately 25.4-35.9% of patients with PNH have thrombosis during the diagnosis [6]. Hepatic veins are the most commonly involved site in PNH, isolated involvement of portal vein with visceral veins without the involvement of the hepatic veins is rare as depicted in our case, and the mortality risk associated with thrombosis in PNH was approximately 25.4%. Given the increased mortality risk, it is important to diagnose early and treat the condition [4].

The mechanism of thrombosis is unclear, although the literature has reported multiple factors associated with thrombosis in PNH, and several theories have been proposed. The first of these is the activation of granulocytes to release inflammatory molecules that damage the endothelium. As a result, leukocytes adhere to the damaged endothelium, which initiates the inflammatory process via inflammatory cytokine release, and the absence of the complement regulatory proteins CD55 and CD59 on the plasma cell membrane in PNH makes these cells more susceptible to complement-mediated activation and disruption [7]. Previous studies have reported a positive correlation between granulocyte clone size and risk of thrombosis [8, 9]. Another proposed mechanism is impaired fibrinolysis caused by a failure to convert plasminogen to plasmin. This is due to the absence of urokinase plasminogen activator on the surface of leukocytes in PNH [10]. Lastly, high levels of hemoglobin from hemolysis can lead to nitric oxide depletion, which was also reported to contribute to thrombosis [11].

PNH patients present with diverse clinical presentations from hemolysis to thrombosis. Abdominal pain, dysphagia, erectile dysfunction, and hematuria are common symptoms in PNH. The former symptoms can be explained by nitric oxide (NO) depletion, while the latter symptoms are due to hemoglobinuria; both occur as a result of elevated hemoglobin from increased intravascular hemolysis. As noted above, thrombosis is another finding in PNH patients. In these cases, laboratory findings can show anemia with an increased reticulocyte count, elevated LDH, elevated indirect bilirubin and decreased haptoglobin from hemolysis and when associated with other bone marrow disorders such as aplastic anemia, hypocellularity, pancytopenia, a decreased reticulocyte count can be seen on peripheral blood smear [12]. Ultrasonography is the initial investigation and the least expensive of all imaging modalities, with an overall sensitivity of 80% to 100%, but it has limited ability to provide additional information such as associated bowel infarction [13]. CT scan and MRI are more sensitive tools used in the diagnosis of venous thrombosis as well as bowel infarction and can reveal the status of adjacent organs [14, 15].

Given its association with increased mortality, the aim of thrombosis management is to treat and prevent its advancement. Anticoagulation has been proven effective in non-cirrhotic acute portal vein thrombosis. A systematic review reported that complete or partial portal vein recanalization occurred in 38.3% and 14% of cases, respectively, after the initiation of anticoagulation compared to 16.7% without anticoagulation [16]. Some authors have reported lifelong anticoagulation in patients with significant prothrombotic risk factors. Based on symptom acuity and presentation, intravenous (IV) heparin can be used with or without vitamin K antagonists. Eculizumab and allogenic bone marrow transplantation are the most widely used treatment modalities for secondary prevention of disease. A humanized anti-C5 monoclonal antibody, eculizumab acts by targeting complement C5, thus inhibiting the formation of membrane-associated complexes on red blood cell membranes and reducing damage. Multiple studies have reported the safety and efficacy of eculizumab in PNH [17-20]. All these studies have shown this drug to be effective in reducing intravascular hemolysis, decreasing the need for red cell transfusions, and mitigating the risk of thrombosis, thus improving quality of life. Bone marrow transplantation (BMT) is an alternative option for patients who have failed eculizumab therapy or in countries where eculizumab is not available.

This article was presented as a poster at American College of Gastroenterology Annual Scientific Meeting on October 14 - 19, 2016 at Las Vegas, NV. 

## Conclusions

Thrombosis is associated with increased mortality in PNH patients. Early diagnosis and awareness of differentials are very important in patients with non-cirrhotic portal vein thrombosis. Early anticoagulation and appropriate treatment can help improve survival in PNH.

## References

[1] (2015). Paroxysmal Nocturnal Hemoglobinuria (PNH) - NORD (National Organization for Rare Disorders). https://rarediseases.org/physician-guide/paroxysmal-nocturnal-hemoglobinuria-pnh/.

[2] Bessler M, Hiken J (2008). The pathophysiology of disease in patients with paroxysmal nocturnal hemoglobinuria. Hematology Am Soc Hematol Educ Program.

[3] Socié G, Mary JY, de Gramont A (1996). Paroxysmal nocturnal haemoglobinuria: long-term follow-up and prognostic factors. Lancet.

[4] Ziakas PD, Poulou LS, Rokas GI, Bartzoudis D, Voulgarelis M (2007). Thrombosis in paroxysmal nocturnal hemoglobinuria: sites, risks, outcome. An overview. J Thromb Haemost.

[5] Ponziani FR, Zocco MA, Campanale C (2010). Portal vein thrombosis: Insight into physiopathology, diagnosis, and treatment. World J Gastroenterol.

[6] De Latour RP, Mary JY, Salanoubat C (2008). Paroxysmal nocturnal hemoglobinuria: natural history of disease subcategories. Blood.

[7] Wiedmer T, Hall SE, Ortel TL, Kane WH, Rosse WF, Sims PJ (1993). Complement-induced vesiculation and exposure of membrane prothrombinase sites in platelets of paroxysmal nocturnal hemoglobinuria. Blood.

[8] Moyo VM, Mukhina GL, Garrett ES, Brodsky RA (2004). Natural history of paroxysmal nocturnal haemoglobinuria using modern diagnostic assays. Br J Haematol.

[9] Gupta SK, Pati HP, Tejomurtula AP, Seth T (2010). PNH clone assessment by flow cytometry and its clinical correlation in PNH and aplastic anemia. J Hematop.

[10] Ploug M, Plesner T, Ronne E (1992). The receptor for urokinase-type plasminogen activator is deficient on peripheral blood leukocytes in patients with paroxysmal nocturnal hemoglobinuria. Blood.

[11] Dusse LMSA, Cooper AJ, Lwaleed BA (2007). Tissue factor and nitric oxide: a controversial relationship!. J Thromb Thrombolysis.

[12] Brodsky RA (2014). Paroxysmal nocturnal hemoglobinuria. Blood.

[13] Van Gansbeke D, Avni EF, Delcour C, Engelholm L, Struyven J (1985). Sonographic features of portal vein thrombosis. Am J Roentgenol.

[14] Lee H-K, Park SJ, Yi B-H, Yeon E-K, Kim JH, Hong H-S (2008). Portal vein thrombosis: CT features. Abdom Imaging.

[15] Jha RC, Khera SS, Kalaria AD (2018). Portal vein thrombosis: imaging the spectrum of disease with an emphasis on MRI features. Am J Roentgenol.

[16] Hall TC, Garcea G, Metcalfe M, Bilku D, Dennison AR (2011). Management of acute non-cirrhotic and non-malignant portal vein thrombosis: a systematic review. World J Surg.

[17] Hillmen P, Muus P, Dührsen U (2007). Effect of the complement inhibitor eculizumab on thromboembolism in patients with paroxysmal nocturnal hemoglobinuria. Blood.

[18] Weitz IC, Razavi P, Rochanda L (2012). Eculizumab therapy results in rapid and sustained decreases in markers of thrombin generation and inflammation in patients with PNH independent of its effects on hemolysis and microparticle formation. Thromb Res.

[19] Hillmen P, Muus P, Röth A (2013). Long-term safety and efficacy of sustained eculizumab treatment in patients with paroxysmal nocturnal haemoglobinuria. Br J Haematol.

[20] Kelly RJ, Hill A, Arnold LM (2011). Long-term treatment with eculizumab in paroxysmal nocturnal hemoglobinuria: Sustained efficacy and improved survival. Blood.

